# Supplementary material for: The Systems Biology Research Tool: evolvable open-source software
Source: BMC Syst Biol. 2008 Jun 29;2:55. doi: 10.1186/1752-0509-2-55 (PMC2446383; doi:10.1186/1752-0509-2-55)
Supplement: Additional file 1 — SBRT Archive. An archive of the current version of the Systems Biology Research Tool. [file 1752-0509-2-55-S1.zip › sbrt-1.4.0/doc/developers_guide/api/sbrt/kernel/fba/comp/FbaOptException.html]

FbaOptException


|  |  |  |
| --- | --- | --- |
| **Overview** \| **Package** \| **Class** \| **Use** \| **Tree** \| **Deprecated** \| **Index** \| **Help** | |  |
| **PREV CLASS**   **NEXT CLASS** | **FRAMES**    **NO FRAMES**     **All Classes** |  |
| SUMMARY: NESTED \| FIELD \| CONSTR \| METHOD | DETAIL: FIELD \| CONSTR \| METHOD |  |


---


## sbrt.kernel.fba.comp Class FbaOptException

```
java.lang.Object
  java.lang.Throwable
      java.lang.Exception
          java.lang.RuntimeException
              sbrt.kernel.fba.comp.FbaOptException
```

**All Implemented Interfaces:**: java.io.Serializable

---

``` public class FbaOptException extends java.lang.RuntimeException ```

This class is used to indicate that an optimal solution could not be found
or does not exist.

**Author:**
:   This class was written and documented by Jeremiah Wright
    while in the Wagner lab.

**See Also:**: Serialized Form

---

**Constructor Summary**

`FbaOptException(MathExpr<?> objectiveFunction,
OptimizationSense sense,
ProgramSolverStatus status)`
  
          Constructs a new FBA optimization exception.

`FbaOptException(MathExpr<?> objectiveFunction,
OptimizationSense sense,
ProgramSolverStatus status,
java.lang.String message)`
  
          Constructs a new FBA optimization exception.


| **Method Summary** | |
| --- | --- |
| `MathExpr<?>` | `getObjFunction()`             Returns the objective function whose optimal value could not be found. |
| `OptimizationSense` | `getSense()`             Returns the sense, or direction, of the attempted optimization. |
| `ProgramSolverStatus` | `getStatus()`             Returns the status of the attempted optimization. |

**Methods inherited from class java.lang.Throwable**

`fillInStackTrace, getCause, getLocalizedMessage, getMessage, getStackTrace, initCause, printStackTrace, printStackTrace, printStackTrace, setStackTrace, toString`

**Methods inherited from class java.lang.Object**

`clone, equals, finalize, getClass, hashCode, notify, notifyAll, wait, wait, wait`

**Constructor Detail**

### FbaOptException

```
public FbaOptException(MathExpr<?> objectiveFunction,
                       OptimizationSense sense,
                       ProgramSolverStatus status)
```

:   Constructs a new FBA optimization exception.

    **Parameters:**: `objectiveFunction` - the objective function whose optimal value could not be found.: `sense` - the sense, or direction, of the attempted optimization.: `status` - the status of the attempted optimization.

---


### FbaOptException

```
public FbaOptException(MathExpr<?> objectiveFunction,
                       OptimizationSense sense,
                       ProgramSolverStatus status,
                       java.lang.String message)
```

:   Constructs a new FBA optimization exception.

    **Parameters:**: `objectiveFunction` - the objective function whose optimal value could not be found.: `sense` - the sense, or direction, of the attempted optimization.: `status` - the status of the attempted optimization.: `message` - a message useful for debugging.


**Method Detail**

### getObjFunction

```
public MathExpr<?> getObjFunction()
```

:   Returns the objective function whose optimal value could not be found.

    :   **Returns:**: the objective function whose optimal value could not be found.

---


### getSense

```
public OptimizationSense getSense()
```

:   Returns the sense, or direction, of the attempted optimization.

    :   **Returns:**: the sense, or direction, of the attempted optimization.

---


### getStatus

```
public ProgramSolverStatus getStatus()
```

:   Returns the status of the attempted optimization.

    :   **Returns:**: the status of the attempted optimization.


---


|  |  |  |
| --- | --- | --- |
| **Overview** \| **Package** \| **Class** \| **Use** \| **Tree** \| **Deprecated** \| **Index** \| **Help** | |  |
| **PREV CLASS**   **NEXT CLASS** | **FRAMES**    **NO FRAMES**     **All Classes** |  |
| SUMMARY: NESTED \| FIELD \| CONSTR \| METHOD | DETAIL: FIELD \| CONSTR \| METHOD |  |


---
